# Retraction: Long non-coding RNA TUG1 promotes cervical cancer progression by regulating the miR-138-5p-SIRT1 axis

**DOI:** 10.18632/oncotarget.28797

**Published:** 2025-12-31

**Authors:** Jie Zhu, Huirong Shi, Huina Liu, Xiaojuan Wang, Fengmei Li

**Affiliations:** ^1^Department of Gynecology and Obstetrics, The First Affiliated Hospital of Zhengzhou University, Zhengzhou 450052, Henan, China; ^2^Department of Obstetrics and Gynecology, Zhengzhou Central Hospital, Zhengzhou 450000, Henan, China

**This article has been retracted:** Oncotarget’s investigation of this paper has concluded. This investigation found that Figure 6B contains a duplicate blot image also found in Figure 5B of an earlier paper [1]. This image also appears in Figure 6 of [2]. Figure 7 duplicates images found in Figure 5C of [2] as well. Both images of mice in Figure 3B are duplicates of those found in Figure 2C of an earlier paper [3]. Oncotarget was also contacted by *Molecular Therapy – Nucleic Acids* journal, whose investigation revealed that there were duplicate images between this article and [4]. Specifically, the transwell assay images in Figure 2D of the MTNA paper, match Transwell assay images in Figure 2E of this Oncotarget paper, and the immunohistochemistry images in Figure 7E of the MTNA paper, match immunohistochemistry images in Figure 3D and 3E of this Oncotarget paper. Corresponding author Jie Zhu has asked for a retraction on behalf of all authors; however, none of the other authors have responded to the journal’s requests for confirmation of the retraction. Based on these facts, the Editorial decision was made to retract this paper.

Original article: Oncotarget. 2017; 8:65253–65264. 65253-65264. https://doi.org/10.18632/oncotarget.18224

## References

[1] Yang FQ , Zhang HM , Chen SJ , Yan Y , Zheng JH . MiR-506 is down-regulated in clear cell renal cell carcinoma and inhibits cell growth and metastasis via targeting FLOT1. PLoS One. 2015; 10:e0120258. 10.1371/journal.pone.0120258. . Corrected in: PLoS One. 2015; 10:e0129404. DOI: 10.1371/journal.pone.0129404 PMID: 26010911 25793370 PMC4368579

[2] Cui Y , Zhang F , Zhu C , Geng L , Tian T , Liu H . Upregulated lncRNA SNHG1 contributes to progression of non-small cell lung cancer through inhibition of miR-101-3p and activation of Wnt/β-catenin signaling pathway. Oncotarget. 2017; 8:17785–94. 10.18632/oncotarget.14854. 28147312 PMC5392286

[3] Gao JZ , Li J , DU JL , Li XL . Long non-coding RNA HOTAIR is a marker for hepatocellular carcinoma progression and tumor recurrence. Oncol Lett. 2016; 11:1791–98. 10.3892/ol.2016.4130. 26998078 PMC4774541

[4] Jiao G , Huang Q , Hu M , Liang X , Li F , Lan C , Fu W , An Y , Xu B , Zhou J , Xiao J . Therapeutic Suppression of miR-4261 Attenuates Colorectal Cancer by Targeting MCC. Mol Ther Nucleic Acids. 2017; 8:36–45. 10.1016/j.omtn.2017.05.010. 28918036 PMC5480279

